# Polyadenylated RNA and RNA-Binding Proteins Exhibit Unique Response to Hyperosmotic Stress

**DOI:** 10.3389/fcell.2021.809859

**Published:** 2021-12-14

**Authors:** Benjamin L. Zaepfel, Jeffrey D. Rothstein

**Affiliations:** ^1^ Biochemistry, Cellular and Molecular Biology Program, Johns Hopkins University School of Medicine, Baltimore, MD, United States; ^2^ Molecular Biology and Genetics Department, Johns Hopkins University School of Medicine, Baltimore, MD, United States; ^3^ Brain Science Institute, Johns Hopkins University School of Medicine, Baltimore, MD, United States; ^4^ Department of Neurology, Johns Hopkins University School of Medicine, Baltimore, MD, United States

**Keywords:** stress granules (SG), sorbitol, osmotic stress, RNA, RNA-binding proteins

## Abstract

Stress granule formation is a complex and rapidly evolving process that significantly disrupts cellular metabolism in response to a variety of cellular stressors. Recently, it has become evident that different chemical stressors lead to the formation of compositionally distinct stress granules. However, it is unclear which proteins are required for the formation of stress granules under different conditions. In addition, the effect of various stressors on polyadenylated RNA metabolism remains enigmatic. Here, we demonstrate that G3BP1/2, which are common stress granule components, are not required for the formation of stress granules specifically during osmotic stress induced by sorbitol and related polyols. Furthermore, sorbitol-induced osmotic stress leads to significant depletion of nuclear polyadenylated RNA, a process that we demonstrate is dependent on active mRNA export, as well as cytoplasmic and subnuclear shifts in the presence of many nuclear RNA-binding proteins. We assessed the function of multiple shifted RBPs and found that hnRNP U, but not TDP-43 or hnRNP I, exhibit reduced function following this cytoplasmic shift. Finally, we observe that multiple stress pathways lead to a significant reduction in transcription, providing a possible explanation for our inability to observe loss of TDP-43 or hnRNP I function. Overall, we identify unique outcomes following osmotic stress that provide important insight into the regulation of RNA-binding protein localization and function.

## Introduction

Nuclear RNA binding proteins (RBPs) perform a broad range of functions to process mRNA from transcription to nuclear export. The proper processing and maturation of mRNA depends on the temporal and spatial availability of the necessary RBPs. The improper regulation of RBPs and maturation of RNA contributes to the physiology of numerous diseases, including amyotrophic lateral sclerosis (Vanderweyde et al., 2017), fragile X mental retardation syndrome (Vanderweyde et al., 2017), and a variety of cancer subtypes (Qin et al., 2020). A recent comprehensive study identified numerous interactions between RBPs and their target RNAs (Van Nostrand et al., 2020). While this work provides significant insight into the localization and interactions of RBPs in an unperturbed cellular environment, it is important to understand how RBPs and RNA respond to stimulus such as cellular stress.

To build upon this work, we set out to determine how RBPs and mRNA respond to cellular stressors that are commonly used in cell culture models. Commonly employed artificial stress include sodium arsenite to model oxidative stress (Ruiz-Ramos et al., 2009), dithiothreitol (DTT) to induce ER stress (Kaufman, 1999), and sorbitol to induce hyperosmotic stress (Bell et al., 2000). All three of these stressors have been demonstrated to induce recruitment of RBPs and RNA to stress granules (SGs) (Burgess et al., 2011; Hans et al., 2020; Child et al., 2021; Matheny et al., 2021), providing a useful model for us to investigate the mechanism by which RBPs and RNA respond to cellular disturbance. We hypothesize that RBPs and RNA may bi-directionally regulate the other’s localization, and therefore function.

To test this hypothesis, we utilize a combination of molecular biology and microscopy techniques to observe the effect of oxidative, ER, and hyperosmotic stress on RBP and RNA localization. We make the surprising observation that hyperosmotic stress leads to a unique nuclear-to-cytoplasmic and NXF1-dependent re-distribution of polyadenylated RNA. Furthermore, nuclear RBPs demonstrate a similar cytoplasmic-directed shift in localization, which leads to significant loss of function in the most shifted RBP. Taken together, we demonstrate a strong connection between the response of both RNA and RBPs to hyperosmotic stress.

## Materials and Methods

### Cell Culture, Treatment, and Transfection

WT and G3BP1/2 KO U2OS cells were cultured in DMEM/F12 (ThermoFisher Scientific 11330032) supplemented with 10% heat-inactivated fetal bovine serum (GenClone 25-514H) and 1% Penicillin-Streptomycin (ThermoFisher Scientific 15140122). HeLa cells were cultured in DMEM (ThermoFisher Scientific 11995-065) supplemented with 10% heat-inactivated FBS and 1% Penicillin-Streptomycin.

For stress experiments, cells were treated with a final concentration of 100 μM sodium arsenite, 2 mM dithiothreitol (DTT), or 400 mM D-Sorbitol, D-Mannitol, or Xylitol in culture media. For experiments involving ISRIB or GSK2606414, cells were pre-treated with 2 μM ISRIB (Tocris 5284) or 5 μM GSK2606414 (Tocris 5107) for 3 h, then the media was exchanged to include the stressors as well as ISRIB or GSK2606414 for 1 h.

For siRNA transfections, RNAiMAX (ThermoFisher Scientific 13778075) was used as directed by manufacturer. Cells were transfected for 2 days with either Non-targeting (Horizon Discovery Biosciences D-001810-10-05) or NXF1 (L-013680-01-0005) SMARTPool siRNA. After 48 total hours with transfection reagent, the media was exchanged for 24 h, then cells were assayed at 72 h following initial transfection.

### Immunofluorescent Staining

Cells were briefly rinsed in 1× PBS (Quality Biological 119-069-131) and fixed in 4% paraformaldehyde (Electron Microscopy Sciences 15714-S) diluted in 1× PBS for 10 min. Cells were permeabilized in 1× PBS with 0.3% Triton for 10 min, then washed three times in 1× PBS for 10 min 10% Normal goat serum (Vector Labs S-1000) in 1× PBS was used to block for 1 h, then primary antibodies (see Table 1) were diluted in 10% Normal goat serum in 1× PBS before application to cells overnight at 4°C. Cells were again washed three times in 1× PBS, then secondary antibodies (see Table 1) were diluted in 10% normal goat serum in 1× PBS before being applied to cells for 1 h at room temperature. Cells were washed once in 1× PBS for 10 min, once with 1x PBS containing 10 μg/ml Hoechst 33342 (Invitrogen H3570) for 10 min, then once with 1x PBS for 10 min. Stained cells were preserved in a 50:50 solution of glycerol and 1x PBS for imaging.

**TABLE 1:** Antibodies and dilutions.

Antibody	Source/Catalog number	Western Blot dilution	IF dilution
Anti-IMP-1	ProteinTech 22803-1-AP	n/a	1:250
Anti-PABPC1	Santa Cruz Biotechnology sc-21318	n/a	1:50
Anti-TIA1	ProteinTech 12133-2-AP	n/a	1:250
Anti-UBAP2L	abcam ab138309	n/a	1:250
Anti-p-eIF2α	Cell Signaling Technologies 9721S	1:2,000	n/a
Anti-eIF2α	R&D Systems AF3997	1:200	n/a
Anti-α-tubulin	Cell Signaling Technologies 2125S	1:3,000	n/a
Anti-hnRNP A2/B1	Santa Cruz Biotechnology sc-53531	1:100	n/a
Anti-hnRNP K	Santa Cruz Biotechnology sc-28380	1:100	n/a
Anti-hnRNP H	Bethyl Laboratories A300-511A	1:1,000	n/a
Anti-hnRNP F	Santa Cruz Biotechnology sc-32309	1:100	n/a
Anti-hnRNP L	Santa Cruz Biotechnology sc-21317	1:100	n/a
Anti-hnRNP R	Sigma-Aldrich HPA026092	1:100	n/a
Anti-hnRNP I	Santa Cruz Biotechnology sc-56701	1:100	n/a
Anti-hnRNP U	Santa Cruz Biotechnology sc-21315	1:100	n/a
Anti-FUS	Bethyl Laboratories A300-302A	1:1,000	n/a
Anti-hnRNP A1	Santa Cruz Biotechnology sc-32301	1:100	n/a
Anti-hnRNP C1/C2	Santa Cruz Biotechnology sc-32308	1:100	n/a
Anti-TDP-43	ProteinTech 10782-2-AP	1:1,000	n/a
Anti-GAPDH	Cell Signaling Technologies 2118S	1:100	n/a
Anti-Lamin B1	abcam ab16048	1:1,000	n/a
Anti-NXF1	abcam ab129160	1:1,000	n/a
Anti-CRM1	Santa Cruz Biotechnology sc-74454	1:100	n/a
Anti-Ran GTPase	BD Bioscience 610341	1:1,000	n/a
Anti-Rabbit HRP	Cell Signaling Technologies 7074S	1:5,000	n/a
Anti-Mouse HRP	Cell Signaling Technologies 7076S	1:5,000	n/a
Anti-Goat HRP	Abcam ab97110	1:5,000	n/a
Anti-Rabbit AF488	Thermo Fisher Scientific A-11034	n/a	1:1,000
Anti-Mouse AF647	Thermo Fisher Scientific A21245	n/a	1:1,000

For 5-bromouridine incorporation, cells were incubated for 1 h with 1 mM 5-bromouridine (Millipore Sigma 850187) concurrently with the stressors noted in Figure 6. Fixation and staining were performed as above.

### Western Blotting

For whole cell lysates, cells were lysed in RIPA buffer (Sigma Aldrich R0278) on ice for 10 min. Lysates were spun at 12,000 rcf for 15 min to remove debris, and the supernatant was transferred to a new Eppendorf tube. Protein concentrations were quantified using the DC protein assay kit (Bio-Rad 5000111) before addition of 6× Laemmli buffer (12% SDS, 50% glycerol, 3% TrisHCl pH 7.0, 10% 2-mercaptoethanol in de-ionized H_2_O, bromophenol blue to color) to a final 1× concentration. 10 μg of protein per sample was loaded onto 4–20% PROTEAN TGX (Bio-Rad 4568093) or Novex 4–12% Tris-Glycine (Invitrogen XP04125BOX) gels, depending on what was available. Protein was transferred to nitrocellulose using Trans-Blot Mini Transfer stacks (Bio-Rad 1704270) and a Trans-Blot Turbo Transfer System (Bio-Rad 1704150). Membranes were blocked in 5% fat-free milk in 1× TBS with 0.1% Tween-20 for 1 h, then primary antibodies (see Table 1) were diluted in 5% fat-free milk in 1× TBS with 0.1% Tween-20 before application to membranes overnight at 4°C. Membranes were washed four times in 1× TBS with 0.1% Tween-20 for 5 min, then secondary antibodies (see Table 1) were diluted in 5% fat-free milk in 1× TBS with 0.1% Tween-20 before application to membranes for 1 h at room temperature. Membranes were again washed four times in 1× TBS with 0.1% Tween-20. Electrochemiluminescent substrate (SuperSignal West Pico PLUS ECL, ThermoFisher Scientific 24580, Immobilon ECL Ultra Western HRP Substrate, Millipore Sigma WBULS0100) was applied to membranes before imaging on an ImageQuant LAS4000 machine. Images were quantified using FIJI (imagej.net). Abundance of proteins of interest were normalized to GAPDH or α-tubulin abundance, as noted in each figure.

### Fluorescence *in situ* Hybridization

Cells were briefly rinsed in 1× PBS then fixed in 10% formaldehyde (Sigma-Aldrich F8775) diluted in 1× PBS for 20 min. Cells were permeabilized in 1× PBS with 0.1% Tween-20 for 10 min, then washed three times in 1× PBS for 10 min and twice in 2× SSC (Sigma-Aldrich) diluted in 1× PBS for 5 min. Cells were pre-hybridized at 42°C in UltraHyb-Oligo (ThermoFisher Scientific AM8663) for 1 h in a humidified chamber. Cy5-labelled oligodT(50) probe (Integrated DNA Technologies) was hybridized at a concentration of 100 nM in UltraHyb-Oligo overnight at 42°C in a humidified chamber. Cells were then washed in 2× SSC, 0.5× SSC, and 0.1× SSC for 20 min each at 42°C in a humidified chamber, with the 0.5× SSC wash also containing 10 μg/ml Hoechst 33342. Stained cells were preserved in a 50:50 solution of glycerol and 1× PBS for imaging.

### Cytoplasm, Nucleoplasm, Chromatin Fractionation

HeLa cells were fractionated using a modified protocol (Conrad et al., 2017). Following respective treatments, cells were removed from a 10 cm plate by applying 5 ml of 0.05% Trypsin-EDTA (Life Technologies 25300054) for 5 min at 37°C. Five millilitres of ice-cold DMEM was added and the cells were transferred to a 15 ml conical tube. Cells were spun at 200 rcf for 5 min at 4°C, the supernatant was aspirated, and the pellet was resuspended in 1 ml of ice-cold 1x PBS and transferred to an Eppendorf tube. Cells were spun at 200 rcf for 2 min at 4°C, then the supernatant was aspirated and 400 μl of lysis buffer (10 mM TrisHCl pH 7.4, 150 mM NaCl, 0.15% IGEPAL) was used to resuspend the pellet by pipetting up and down six times. Cells were incubated for 5 min on ice, then the lysate as carefully layered over 1 ml of sucrose buffer (10 mM TrisHCl, 150 mM NaCl, 24% sucrose) in a new Eppendorf tube. This was spun at 3,500 rcf for 10 min at 4°C. 200uL of the supernatant was carefully removed from the top, and this was used as the cytoplasmic fraction after addition of 40 µl of 6× Laemmli. The rest of the supernatant was aspirated, and the nuclear pellet was rinsed carefully with 1 ml ice-cold 1x PBS, which was also aspirated. The nuclear pellets were resuspended in 250 µl glycerol buffer (20 mM TrisHCl pH 7.4, 75 mM NaCl, 0.5 mM EDTA, 50% glycerol) before quickly adding 250 µl of urea buffer (10 mM TrisHCl pH 7.4, 1 M urea, 0.3 M NaCl, 7.5 mM MgCl_2_, 0.2 mM EDTA, 1% IGEPAL) and vortexing for 4 s. Cells were then incubated one ice for 2 min. Cells were spun at 13,000 rcf for 2 min at 4°C. Two hundred microlitres of supernatant was carefully taken as the nucleoplasmic fraction after addition of 40 µl 6× Laemmli. The rest of the supernatant was aspirated, and the chromatin pellet was rinsed in 1 ml of ice-cold 1× PBS. This was aspirated, and 200 µl of 1× Laemmli was added. This chromatin fraction was then passed through a 20-gauge needle 15 times, followed by passage through a 26-gauge needle 10 times. For western blot analysis, 8 µl of each fraction was run, as above.

### RNA Isolation and qRT-PCR Analysis

RNA was isolated with TRIzol (ThermoFisher Scientific 15-596-018) as per manufacturer’s provided protocol. cDNA was synthesized from 1 μg of total RNA using the High-Capacity cDNA Reverse Transcription kit (Fisher Scientific 4368814). qRT-PCR was performed in technical triplicates using primers designed against the described targets (see Table 2). Twenty nanograms of cDNA was loaded into each well of a 96-well plate (Thermo Fisher Scientific 4346907) with 500 nM forward and reverse primers, 5 µl Fast SYBR Green Master Mix (Thermo Scientific 4385612), and nuclease-free H_2_O up to 10 µl. Reactions were performed using a QuantStudio 3 Real-Time PCR System.

**TABLE 2:** qRT-PCR primer sequences.

Target gene	Forward primer sequence (5′→3′)	Reverse primer sequence (5′→3′)	Source
ZNF565	GAC​GAT​GGA​GCT​CTT​GAG​GAC	CCA​CGT​CCC​TGA​ATG​TCA​CC	This paper
GSTM4	GGT​ACT​GGG​ACA​TCC​GCG​G	CAG​CCA​CTG​GCT​TCT​GTC​A	This paper
DOCK1	AGCGCGAGGAGAAGTACG	CCT​CGG​TAC​CAC​CCT​TCA​TA	This paper
PFKP	GCGGGGATGCTCAAGGT	CGT​CCA​CCA​TGC​CCT​GGT​AG	This paper
Unc13A	GGA​CGT​GTG​GTA​CAA​CCT​GG	GTG​TAC​TGG​ACA​TGG​TAC​GGG	Ma et al. (2021)
PTBP2	CGG​TTC​TTG​TGA​GCG​AAG​CT	CAC​TGC​CTG​AGA​GTA​GTT​CGT​C	This paper
IER3	ACCGAAAGCGCAGCCG	CGA​TGG​TGA​GCA​GCA​GAA​AG	This paper
GAPDH	GAA​GGT​GAA​GGT​CGG​AGT​C	GAA​GAT​GGT​GAT​GGG​ATT​TC	Melamed et al. (2019)

### Imaging and Data Analysis

Four to nine non-overlapping fields of view with ×40 magnification were imaged using an ImageXpress Micro XLS high-content microscope. For the RNA FISH analysis, nuclear and cytoplasmic fluorescence intensity was calculated using a built-in MetaXpress “translocation-enhanced” module. The quotient of these values for each cell was then used as the NC ratio. For BrU analysis, nuclear BrU intensity was determined using Hoechst signal as a mask with the built-in MetaXpress “Find Blobs” module, and the average nuclear BrU intensity from the untreated wells was used as the normalizing factor for nuclear BrU intensity from the stressor-treated wells.

## Results

### G3BP1/2 are not Required for Stress Granule Formation During Hyperosmotic Stress

Both G3BP1 and G3BP2 have both been demonstrated as resident protein components of SGs that form in response to cellular stress (Matsuki et al., 2013). Recently, it has been observed that in U2OS cells, SGs are still observed even with the G3BP1 and G3BP2 genes both knocked out (G3BP1/2 KO), some SGs are still observed (Kedersha et al., 2016; Yang et al., 2020). This suggests that multiple different proteins are sufficient to induce the formation of SGs, and perhaps that G3BP1/2 are not wholly responsible for the recruitment of RNA and other RBPs to these cytoplasmic puncta.

**FIGURE 1:**
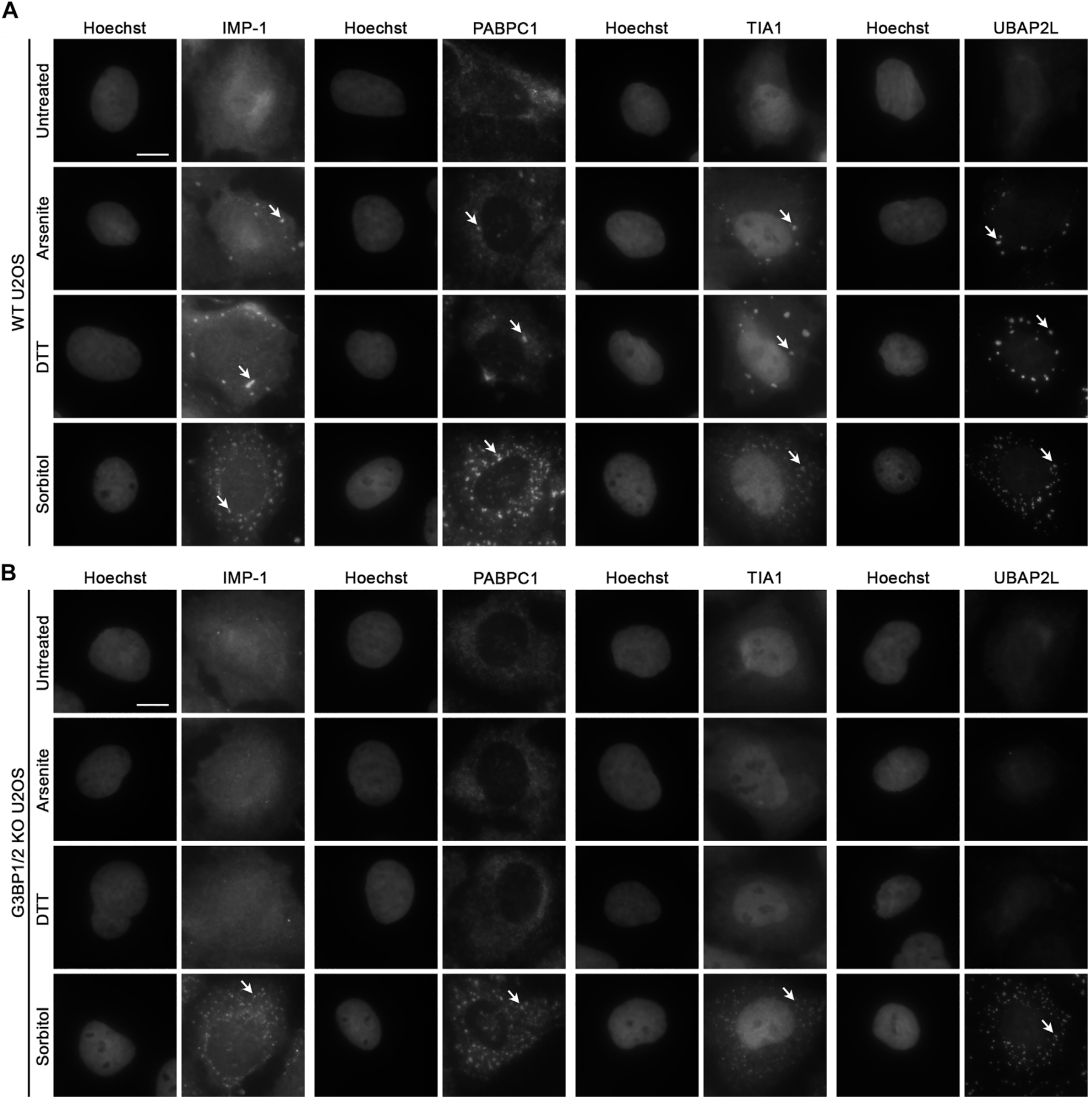
Recruitment of RBPs to hyperosmotic stress-induced granules does not require G3BP1/2. Representative images of **(A)** WT and **(B)** G3BP1/2 KO U2OS cells stained for various stress granule resident RNA-binding proteins (IMP-1, PABPC1, TIA1, and UBAP2L). Cells were treated with 100 μM sodium arsenite, 2 mM DTT, or 400 mM D-sorbitol for 1 h as indicated. Arrows indicate accumulation of each RBP in stress granules. Images are representative of three independent biological replicates that yielded similar results. The scale bar represents 5 μm.

To further probe whether the loss of G3BP1/2 affects recruitment of other known SG components, we treated wild-type (WT) and G3BP1/2 KO U2OS with 100 μM sodium arsenite, 2 mM DTT, or 400 mM D-sorbitol for 1 h to induce oxidative, ER, and hyperosmotic stress, respectively. In WT cells, each stressor led to the presence of punctate immunofluorescence (IF) signal in the cytoplasm for four different SG components (IMP-1, PABPC1, TIA1, and UBAP2L). The punctate, versus diffuse, signal is how we assess and categorize the formation of SGs in each experiment (Figure 1A; Supplementary Figure S1). The SGs formed following sorbitol treatment are more numerous than those formed following arsenite or DTT treatment (Supplementary Figure S2).

In G3BP1/2 KO cells treated similarly, neither sodium arsenite nor DTT lead to incorporation of any of the tested SG components into SGs (Figure 1B). However, sorbitol leads to the formation of SGs in G3BP1/2 KO cells that are similar in appearance to those formed in WT cells under the same condition (Figures 1A,B; Supplementary Figure S1). Thus, neither G3BP1 nor G3BP2, in spite of their participation in SG dynamics under some stress conditions, are required for the formation of SGs during sorbitol-induced hyperosmotic stress.

### eIF2α Activity is not Required for Stress Granule Formation During Hyperosmotic Stress

eIF2α protein is highly involved in the integrated stress response (ISR) (Pakos-Zebrucka et al., 2016). As the cell responds to various forms of cellular stress, eIF2α is phosphorylated by one of four kinases: PERK (Prostko et al., 1993), HRI (Chen et al., 1991), PKR (Clemens and Elia, 1997), and GCN2 (Vazquez de Aldana et al., 1994). Phosphorylated eIF2α (p-eIF2α) then functions to reduce cap-dependent mRNA translation and induce downstream transcriptional alterations via ATF4 (Pakos-Zebrucka et al., 2016). Recently, compounds have been identified that either reduce the phosphorylation of eif2α or p-eIF2α activity (GSK2606414 and ISRIB, respectively) (Axten et al., 2012; Sidrauski et al., 2015). This in turn prevents ISR-induced SG formation.

**FIGURE 2:**
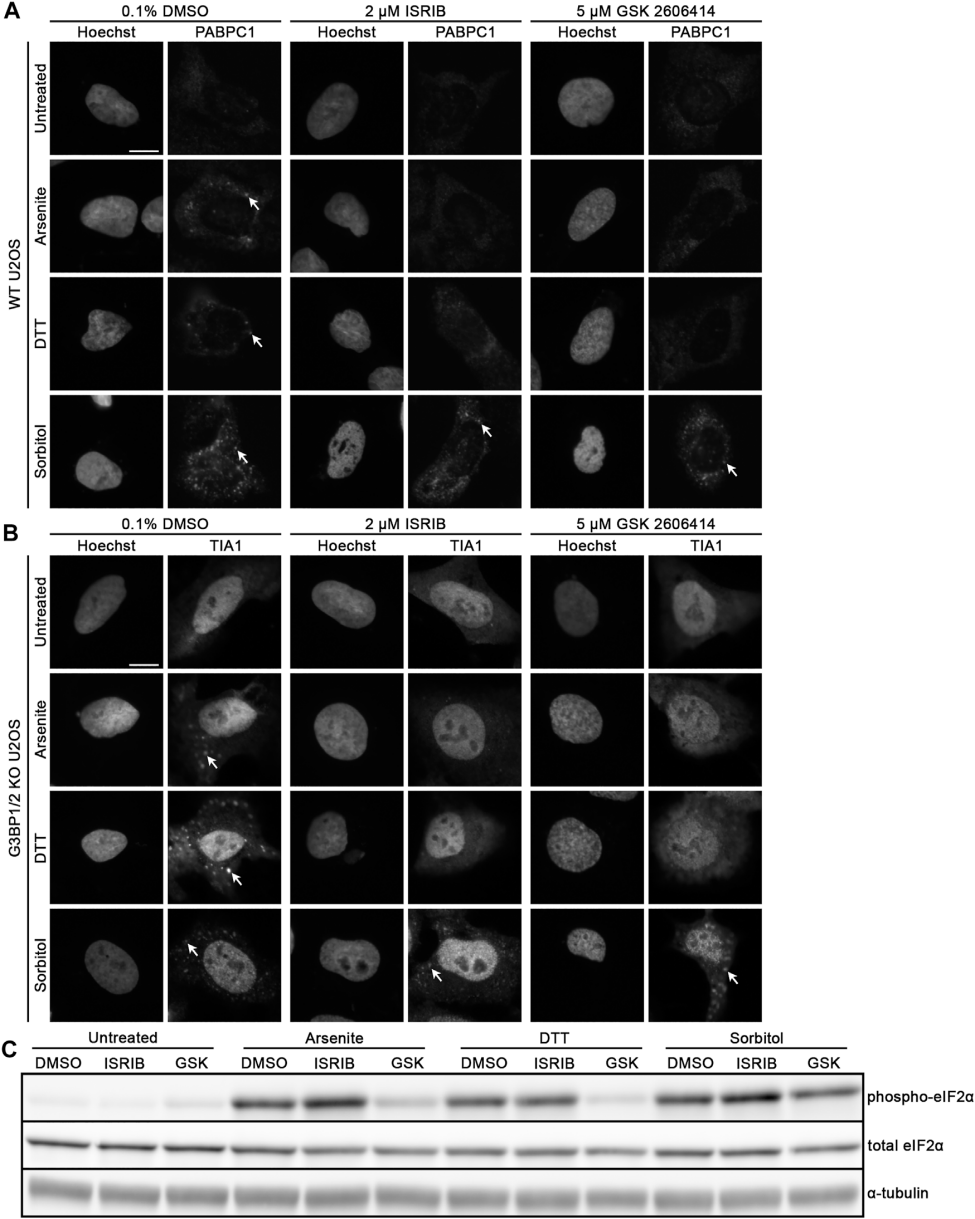
Hyperosmotic stress induces cytoplasmic granule formation independent of canonical integrated stress response pathway. Representative images of WT U2OS cells stained for **(A)** PABPC1 and **(B)** TIA1. Cells were pre-treated with the indicated lead compounds (0.1% DMSO, 2 μM ISRIB, or 5 μM GSK2606414) for 3 h, then co-treated with those chemicals and the indicated (left) stressors for 1 h. Arrows indicate accumulation of each RBP in stress granules. Images are representative of three independent biological replicates that yielded similar results. **(C)** Western blot for p-eIF2α, total eIF2α, and α-tubulin following treatment of WT U2OS cells as in **(A,B)**. The presented images are representative of three independent biological replicates that yielded similar results. The scale bar represents 5 μm.

Since we observed that G3BP1/2 are not required for SG formation following sorbitol-induced hyperosmotic stress, we then wondered whether p-eIF2α and its function are similarly dispensable, as was recently observed by others in the context of TDP-43 hyperphosphorylation (Hans et al., 2020). We treated WT U2OS cells with 0.1% DMSO, 2 μM ISRIB, or 5 μM GSK2606414 for 3 h, then exchanged the media to include sodium arsenite, DTT, or sorbitol in addition to the same chemicals from pre-treatment. PABPC1 (Figure 2A; Supplementary Figure S1) and TIA1 (Figure 2B; Supplementary Figure S1) demonstrated punctate cytoplasmic localization in cells treated with DMSO and a stressor, similar to what we observed in Figure 1. Also as expected, cells pretreated with ISRIB or GSK2606414 and sodium arsenite or DTT no longer formed PABPC1+ nor TIA1+ SGs (Figures 2A,B). In contrast, sorbitol-treated cells formed similar SGs whether they were treated with DMSO, ISRIB, or GSK2606414 (Figures 2A,B; Supplementary Figure S1). Surprisingly, we observed that while sorbitol treatment does induce phosphorylation of eIF2α, GSK2606414 treatment does alter the ratio of phospho-eIF2α to total eIF2α, despite significantly reducing this ratio in the context of sodium arsenite or DTT treatment as determined by Western blot (Figure 2C; Supplementary Figure S3).

### Hyperosmotic Stress Induces Nuclear Clearance of Polyadenylated RNA

**FIGURE 3:**
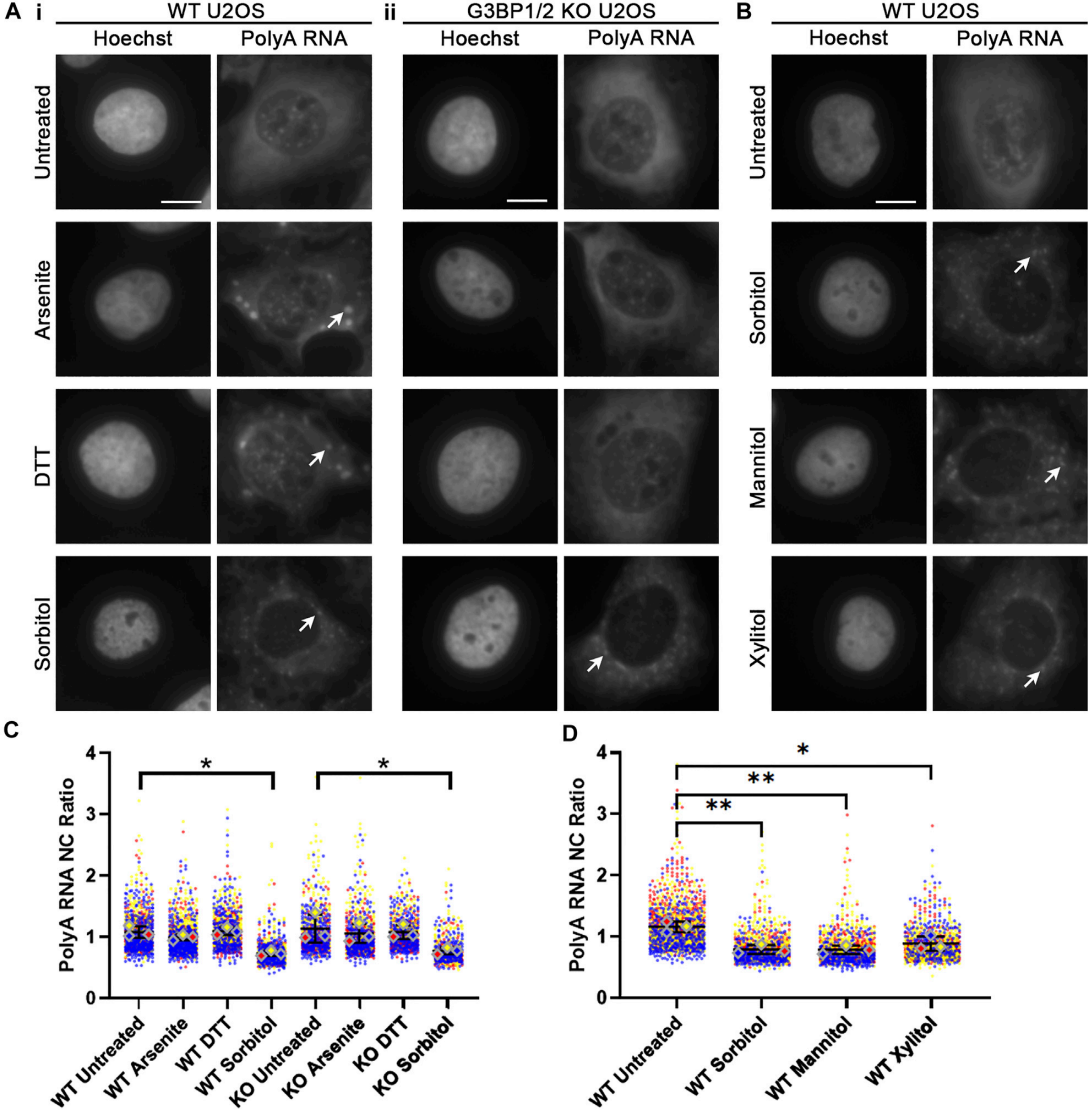
Hyperosmotic stress leads to reduction of nuclear polyadenylated RNA. **(A)** Representative images of WT **(i)** and G3BP1/2 KO **(ii)** U2OS cells stained for polyA RNA using oligodT(50) probes. Cells were treated with the indicated stressors for 1 h. Arrowheads indicate accumulation of polyA RNA in stress granules. Images are representative of three independent biological replicates. **(B)** Representative images of WT U2OS cells stained for polyA RNA following treatment for 1 h with 400 mM of the indicated polyols. Arrowheads indicate accumulation of polyA RNA in stress granules. **(C,D)** Quantification of NC ratio of polyA RNA in each individual cell represented in **(A,B)**. Small circles represent the polyA RNA NC ratio of individual cells, while large diamonds represent the average of the NC ratio of cells from each replicate. *N* = 3 biological replicates with >50 cells per replicate. Ordinary one-way ANOVA was used to calculate statistical significance on the average values from each replicate. *p < 0.05, **p < 0.01. The scale bar represents 5 μm.

Given the crucial role that RNA plays in nucleating SG formation following treatment of cells with sodium arsenite (Bounedjah et al., 2014), we next considered whether polyadenylated (polyA) RNA is in fact present within sorbitol-induced SGs. WT and G3BP1/2 KO U2OS cells were treated as in Figure 1, then polyA RNA was visualized via FISH using a Cy5-labelled oligodT(50) probe (Figure 3A). PolyA RNA was observed to localize to SGs in WT U2OS following treatment with sodium arsenite, DTT, and sorbitol, but only following sorbitol treatment in G3BP1/2 KO cells (Figure 3A). Interestingly, we observed that sorbitol, but not sodium arsenite nor DTT treatment, leads to a significant decrease in the nuclear-to-cytoplasmic (NC) ratio of polyA RNA in both WT and G3BP1/2 KO U2OS cells (Figures 3A,C). Furthermore, when we performed this experiment with the same concentration of two other polyols (D-Mannitol and Xylitol), we observed similar changes to the NC ratio of polyA RNA (Figures 3B,D). These observations suggest that hyperosmotic stress induces this change, rather than an off-target effect of sorbitol treatment itself. Importantly, the sorbitol-dependent change in polyA NC ratio occurs with no significant change in the abundance of proteins intimately involved in the mRNA export pathways, NXF1 and CRM1, or RanGTPase, a protein central to the nuclear import/export pathway (Supplementary Figure S4).

### NXF1 Knockdown Prevents Sorbitol-Induced Egress of Nuclear polyA RNA

**FIGURE 4:**
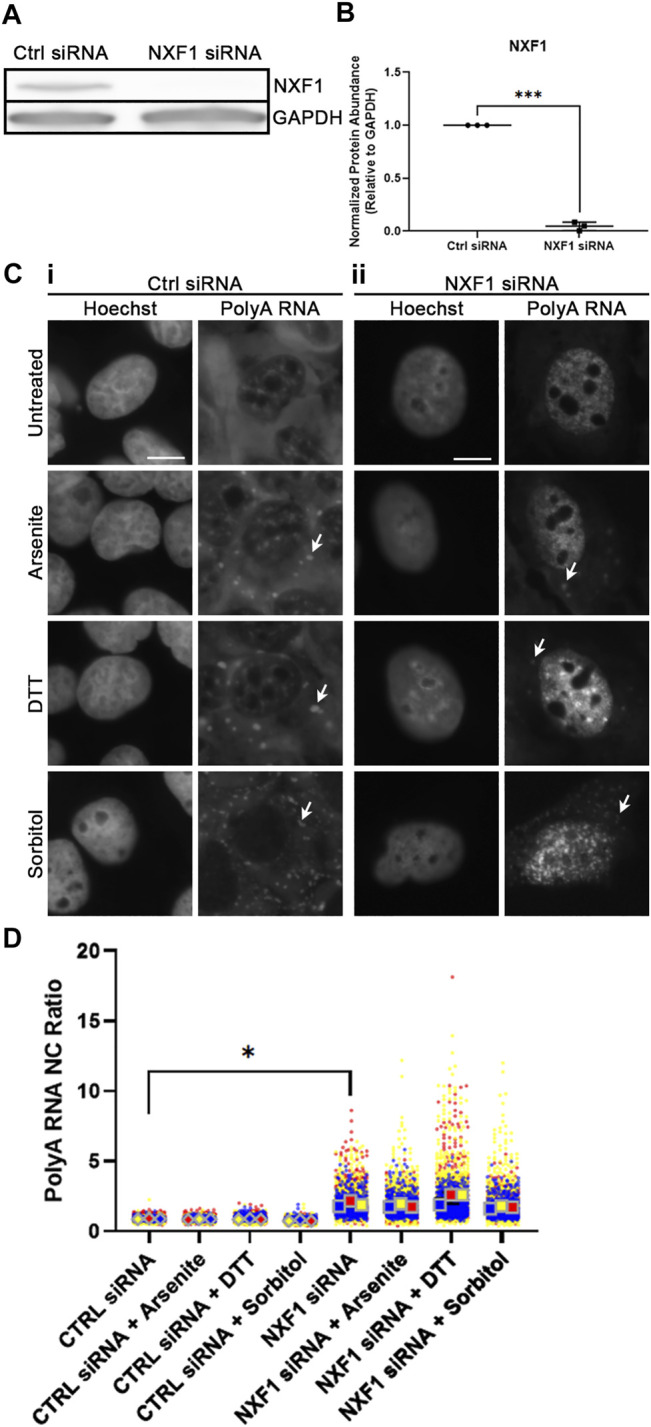
NXF1 regulates sorbitol-induced nuclear polyA RNA egress. **(A)** Western blot of WT U2OS whole cell lysates transfected with Ctrl or NXF1 siRNA. Images are representative of three independent biological replicates. **(B)** Quantification of NXF1 intensity relative to GAPDH intensity from the Western blots represented in **(A)**. NXF1 siRNA values are normalized to the Ctrl siRNA values within the corresponding replicate. Student’s Paired *t*-test was used to calculate statistical significance. **(C)** Representative images of WT U2OS cells transfected with Ctrl siRNA **(i)** or NXF1 siRNA **(ii)** and stained for polyA RNA. Cells were treated with the indicated stressors for 1 h. Arrowheads indicate accumulation of polyA RNA in stress granules. **(D)** Quantification of NC ratio of polyA RNA in each individual cell represented in **(C)**. Small circles represent the polyA RNA NC ratio of individual cells, while large diamonds and squares represent the average of the NC ratio of cells from each replicate. *N* = 3 biological replicates with >50 cells per replicate. Ordinary one-way ANOVA was used to calculate statistical significance on the average values from each replicate. *p < 0.05, ***p < 0.001. The scale bar represents 5 μm.

Following our observation that polyol-induced hyperosmotic stress leads to a significant decrease in the NC ratio of polyA RNA (Figure 3), we wanted to know whether this was a result of active mRNA export. To test this, we knocked down NXF1, one of the protein factors required for the major mRNA export pathway (Carmody and Wente, 2009). Using siRNA, we transfected WT U2OS cells with either a non-targeting or NXF1-targeting SMARTPool of four siRNAs for 48 h, then replaced with normal media for another 24 h. This led to significant reduction in the total abundance of NXF1 (Figures 4A,B).

Next, we stressed WT U2OS cells that had been transfected as above with sodium arsenite, DTT, or sorbitol for 1 h, then analyzed the NC ratio of polyA RNA via FISH. We observed that knockdown of NXF1 leads to a significant increase in the retention, and therefore NC ratio, of polyA RNA (Figures 4C,D). This nuclear retention persisted in all conditions, even following sorbitol-induced hyperosmotic stress (Figures 4C,D). This signifies that the reduced NC ratio of polyA RNA following sorbitol treatment (Figure 3D) is likely a result of active (NXF1-dependent) mRNA export.

### Sorbitol Treatment Induces Subcellular Shift in Numerous RNA-Binding Proteins

Since we observed that sorbitol-induced hyperosmotic stress leads to a significant change in the NC ratio of polyA RNA, we decided to assess the effect of sorbitol treatment on the localization of RNA-binding proteins (RBPs). Sorbitol has previously been shown to induce shifts in proteins such as TDP-43 (Dewey et al., 2011; Wobst et al., 2017) and hnRNP A1 (Courteau et al., 2015). These previous groups performed nucleocytoplasmic fractionations and Western blotting or IF to observe sorbitol-induced changes in RBP localization.

**FIGURE 5:**
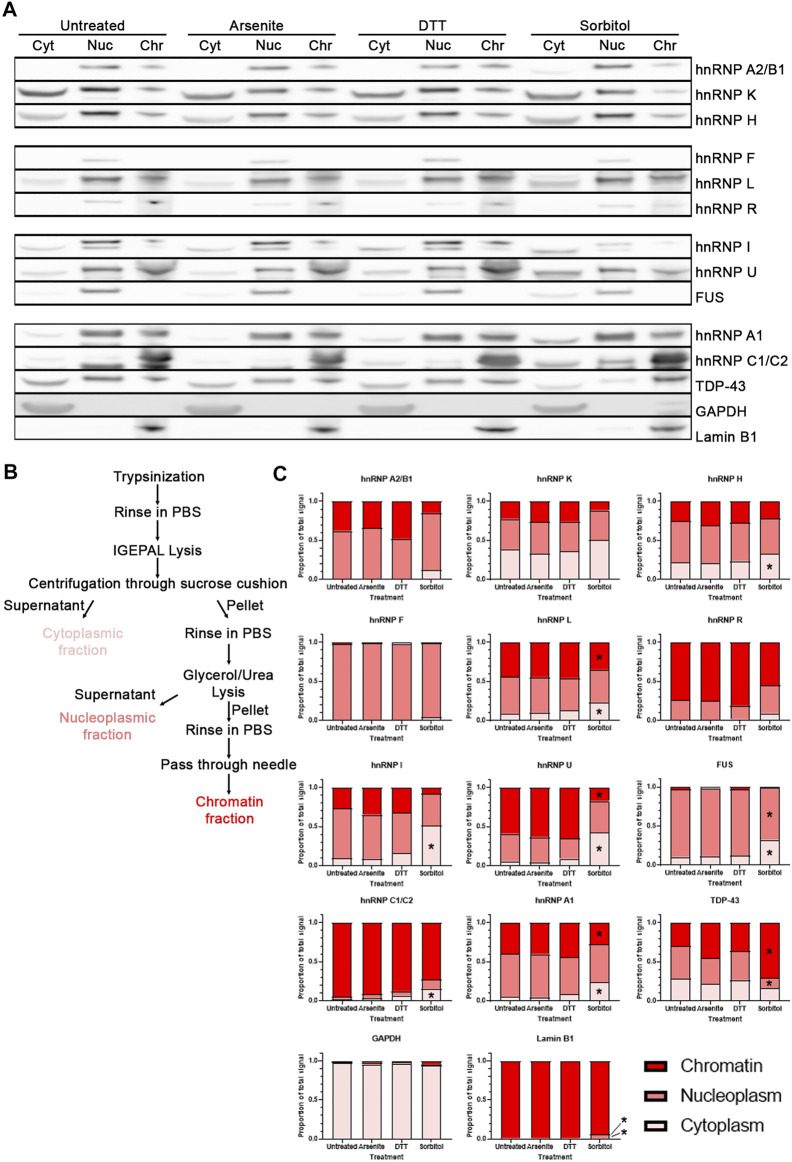
Hyperosmotic stress induces subcellular shift in localization of RBPs. **(A)** Western blots of HeLa cells fractionated into cytoplasmic (Cyt), nucleoplasmic (Nuc), and chromatin-associated (Chr) fractions. Images are all presented from the same biological replicate, grouped based on the probes for each distinct membrane (4 gels/membranes were needed for each replicate). A total of three biological replicates were obtained and probed for each of the indicated proteins. **(B)** Schematic of the isolation protocol used to obtain the samples from **(A)**. **(C)** Quantification of the relative proportion of each RBP within the three fractions. The intensity of the bands from each fraction were summed, and the intensity of the band from each fraction was divided by this sum to determine the relative proportion of the proteins within each fraction by sample. Two-way ANOVA was used to calculate statistical significance. * indicates that the noted fraction contains a significantly different proportion of the protein in the stressed sample relative to the untreated sample. Precise *p* values are presented in Table 3.

Although translocation of proteins from the nucleus to cytoplasm leads to alterations in function, subnuclear changes in localization may also change protein behavior. To delve deeper into how hyperosmotic stress may affect the subnuclear localization of RBPs, we modified a fractionation method that allows for separation of cytoplasm, nucleoplasm, and chromatin-bound proteins (Figure 5B; Conrad et al., 2017). HeLa cells were used for this experiment as U2OS cells could not be appropriately fractionated using this protocol. We treated HeLa cells as in Figure 1, then fractionated each sample and analyzed protein localization via Western blotting (Figure 5A). We verified efficient separation of the fractions by blotting for a cytoplasmic-specific protein (glyceraldehyde-3-phosphate dehydrogenase, GAPDH) and a chromatin-specific protein (Lamin B1). We then probed these blots for a variety of heterogeneous nuclear ribonucleoproteins (hnRNPs), since they comprise a generally well-characterized family of proteins with primarily nuclear localization, as well as two other RBPs of interest, FUS and TDP-43.

**TABLE 3:** Figure 5 adjusted *p*-values.

Treatment	Protein of interest	Fraction	Adjusted *p*-value
Sorbitol	hnRNP A1	Cytoplasm	<0.0001
Sorbitol	hnRNP A1	Chromatin	0.0058
Sorbitol	TDP-43	Nucleoplasm	0.0090
Sorbitol	TDP-43	Chromatin	0.0002
Sorbitol	Lamin B1	Nucleoplasm	0.0001
Sorbitol	Lamin B1	Chromatin	<0.0001
Sorbitol	hnRNP H	Cytoplasm	0.0361
Sorbitol	hnRNP L	Cytoplasm	<0.0001
Sorbitol	hnRNP L	Chromatin	0.0066
Sorbitol	hnRNP I	Cytoplasm	0.0008
Sorbitol	hnRNP U	Cytoplasm	<0.0001
Sorbitol	hnRNP U	Chromatin	<0.0001
Sorbitol	FUS	Cytoplasm	0.0003

We quantified the relative proportion of each RBPs within these fractions and determined that treatment of cells with sorbitol, but not sodium arsenite nor DTT, leads to a significant shift in the subcellular localization of a subset of the RBPs tested (Figure 5C). Specifically, we observe that hnRNP H, hnRNP L, hnRNP I, hnRNP U, FUS, hnRNP C1/C2, and hnRNP A1 become significantly more cytoplasmic following sorbitol treatment. At the same time, hnRNP L, hnRNP U, and hnRNP A1 exhibit significantly decreased association with the chromatin fraction, while FUS decreases in association with the nucleoplasm. TDP-43, which can become more cytoplasmic following sorbitol treatment (Dewey et al., 2011; Wobst et al., 2017), exhibits a tighter association with the chromatin and a corresponding decreased association with the nucleoplasmic fraction. Notably, these changes in localization occur without significant alterations to the abundance of the tested proteins (Supplementary Figure S4). Ultimately, we observe that sorbitol induces a shift in the localization of multiple RBPs that neither sodium arsenite nor DTT mimic.

### hnRNP U but not TDP-43 or hnRNP I Function is Lost Following Hyperosmotic Stress

TDP-43 has been exhaustively shown to localize to SGs upon treatment with sodium arsenite (Liu-Yesucevitz et al., 2010; Dewey et al., 2011; Dammer et al., 2012). We wanted to probe whether observed loss of nuclear TDP-43 function is a consequence of TDP-43 sequestration in SGs. We reviewed two RNA sequencing datasets that describe transcriptomic alterations following TDP-43 knockdown in SH-SY5Y (Melamed et al., 2019) or induced pluripotent stem cell derived human motor neurons (Klim et al., 2019). From these, we identified a subset of RNAs that were upregulated (*ZNF565*, *GSTM4*) or downregulated (*DOCK1*, *PFKP*, *Unc13A*) in both datasets following TDP-43 knockdown. If any particular stress leads to significant loss of TDP-43 function, we would anticipate that this subset of genes would be up- or downregulated accordingly. In the same vein, mRNA targets of hnRNP I (PTBP2) and hnRNP U (IER3) were identified as readouts of their nuclear function (Yugami et al., 2007; Linares et al., 2015).

**FIGURE 6:**
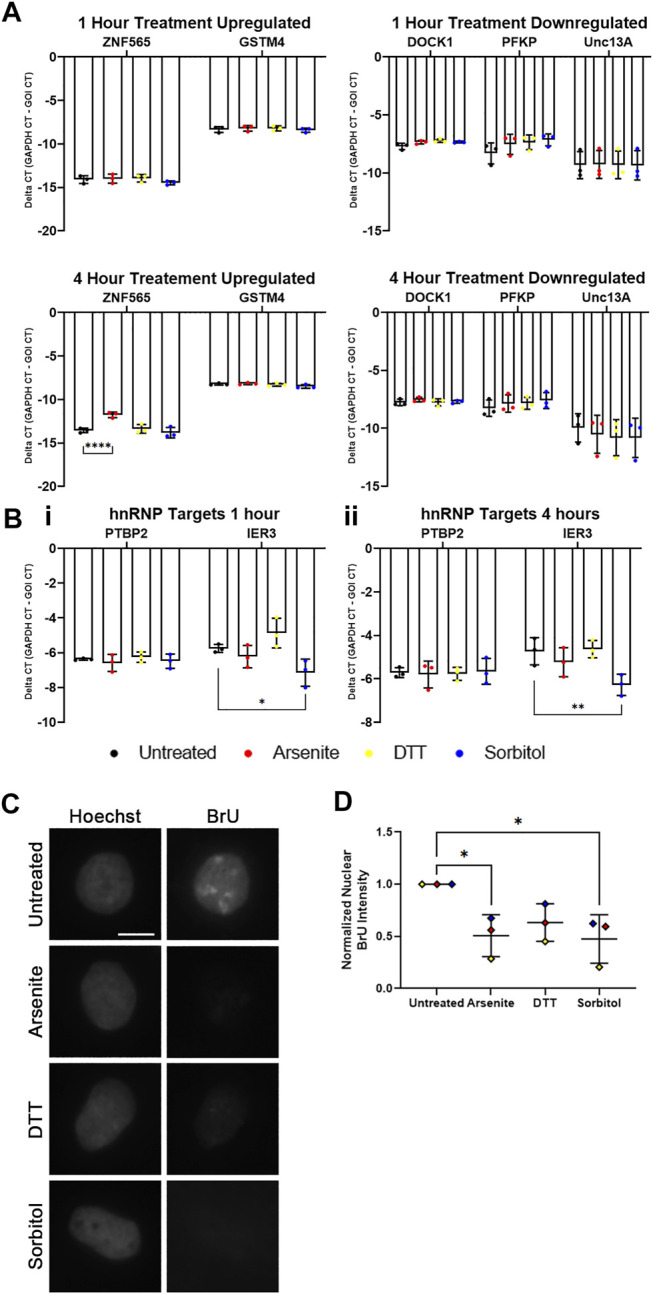
hnRNP U, but not other RBPs, exhibits lost nuclear function following hyperosmotic stress. **(A)** Delta CT of TDP-43 mRNA targets in WT U2OS cells treated with the indicated stressors for 1 h (top) and 4 h (bottom). *ZNF565* and *GSTM4* mRNAs (left) are expected to increase in abundance with lost TDP-43 function, while *DOCK1*, *PFKP*, and *Unc13A* mRNAs (right) are expected to decrease in abundance with lost TDP-43 function. *N* = 3 biological replicates. Two-way ANOVA was used to calculate statistical significance. **(B)** Delta CT of hnRNP mRNA targets in WT U2OS cells treated with the indicated stressors for 1 h **(i)** and 4 h **(ii)**. *PTBP2* mRNA (hnRNP I target) is expected to increase in abundance with lost hnRNP I function. *IER3* mRNA (hnRNP U target) is expected to decrease in abundance with lost hnRNP U function. *N* = 3 biological replicates. Two-way ANOVA was used to calculate statistical significance. **(C)** Representative images of WT U2OS cells stained for incorporated BrU (using a BrdU antibody) following co-treatment with the indicated stressors and 1 mM 5-bromo-uridine for 1 h. **(D)** Quantification of normalized nuclear BrU intensity from images represented in **(C)**. Each data point represents the average nuclear intensity of BrU fluorescence from >50 cells, normalized to the average nuclear BrU intensity from the untreated cells in each biological replicate. *N* = 3 biological replicates. One-way ANOVA was used to calculate statistical significance. *p < 0.05, **p < 0.01, ****p < 0.001. GOI, gene of interest. The scale bar represents 5 μm.

We treated WT U2OS cells with sodium arsenite, DTT, or sorbitol for 1 h, then isolated total RNA and subsequently synthesized cDNA. Using exon-exon junction spanning primer sets designed against each mRNA noted above, we performed qRT-PCR analysis. After 1 h of treatment with the various stressors, we observed no significant changes in the abundance of any TDP-43 or hnRNP I target mRNAs compared to *GAPDH* mRNA (Figures 6A,B). Interestingly, the hnRNP U target *IER3* mRNA is decreased in abundance following sorbitol treatment (Figure 6B). Since 1 h treatment may not be long enough to observe significant changes in the TDP-43 or hnRNP I targets, we repeated the experiment following 4 h of treatment with each stressor. While *ZNF565* mRNA was significantly upregulated after 4 h of sodium arsenite treatment, the other TDP-43 and hnRNP I target mRNAs were unaltered (Figures 6A,B). IER3 remained lower in abundance following this 4-h treatment (Figure 6B). Although more prolonged treatment could lead to more significant changes in these mRNA targets, we observe significant cell death after 5–6 h of treatment with each stressor (data not shown), which would complicate our interpretation of any resulting data.

Since these readouts of RBP function are dependent on transcription, we next wondered whether cellular stress leads to significant alterations in global transcription. KCl-induced hyperosmotic stress is known to reduce global transcription (Rosa-Mercado et al., 2021). Altered transcription could explain why we are unable to observe alterations in TDP-43 or hnRNP I mRNA targets during stress, despite significant cytoplasmic shifting of hnRNP I and localization of TDP-43 to SGs. To quantify the accumulation of nascent RNA during cellular stress, we co-treated WT U2Os cells with 5-bromo-uridine (BrU), a uridine analog that incorporates into nascent RNA and can be visualized via IF using a BrdU antibody (Koberna et al., 2000), with sodium arsenite, DTT, or sorbitol for 1 h. Quantification of the resulting nuclear BrU intensity demonstrated a statistically significant decrease in BrU-labelled RNA following sodium arsenite and sorbitol treatment, and a trending, but not significant, decrease following treatment with DTT (Figures 6C,D). This supports that transcription is broadly reduced during these forms of cellular stress, which is perhaps unsurprising given the major impact of stress on other cellular processes. Furthermore, it provides a possible explanation for why we are unable to find evidence for lost TDP-43 or hnRNP I function during cellular stress.

## Discussion

Altered function and localization of RNA-binding proteins is a well-recognized pathogenic cascade underlying neurodegeneration (Conlon and Manley, 2017; Coyne et al., 2017; De Conti et al., 2017; Maziuk et al., 2017; Zaepfel and Rothstein, 2021), cancer (Wurth, 2012; Kang et al., 2020; Qin et al., 2020), and neurological development (Klein et al., 2016; Prashad and Gopal, 2021; Schieweck et al., 2021). Recent methodological advances have allowed for the generation of immense data sets that describe the binding sites of RBPs on RNA (eCLIP) (Van Nostrand et al., 2016), localization of RNA within tissue (spatial transcriptomics) (Ståhl et al., 2016), and subcellular compartmentalization of RBPs (Lundberg and Borner, 2019). Consequently, many investigations have been focused on understanding the steady-state localization of RBPs, as well as their target mRNAs and protein bindings partners, in unperturbed states (Van Nostrand et al., 2020).

To build upon these big-data investigations, we use a variety of molecular biology techniques to probe the effect of widely used, though non-physiologic, cellular stressors on the localization of specific RBPs and polyA RNA. We broadly validate previous observations that hyperosmotic stress induces formation of SGs independent of G3BP1/2 (Kedersha et al., 2016; Yang et al., 2020; Figure 1) and phospho-eIF2α activity (Hans et al., 2020; Figure 2). The granules formed by sorbitol treatment are more numerous than those formed following sodium arsenite and DTT treatment, and they appear smaller, although this difference in size is not statistically significant (data not shown). This change in number and apparent size of sorbitol-induced granules could be a biophysical effect resulting from altered water abundance in the cells, but this has not been tested. While both sodium arsenite and DTT-induced SGs require the core SG proteins G3BP1/2 to form, sorbitol-induced granules seem largely equivalent when comparing WT and G3BP1/2 KO U2OS cells (Figure 1). This suggests that there exists some cellular factor that nucleates SG formation during hyperosmotic stress, but that is not present or functional during other forms of stress.

It should be noted that although the stressors employed in this study are commonly used to understand cell biological events, the majority are non-physiological and do not reflect natural cellular stressors, except perhaps sorbitol-induced hyperosmotic stress. Mutations in *SORD* cause a subset of Charcot-Marie-Tooth disease, a hereditary neuropathy (Cortese et al., 2020). Loss of function of the SORD enzyme leads to increased plasma concentrations of sorbitol (Cortese et al., 2020). Future investigations into the potential connection between increased plasma sorbitol and long-term hyperosmotic stress will provide insight into the use of sorbitol in truly physiological models of cellular stress.

In addition to the lack of dependence on G3BP1/2 for SG formation during hyperosmotic stress, we observe that neither ISRIB nor GSK2606414 contribute to any noticeable change in the formation of sorbitol-induced SGs (Figure 2A). ISRIB, which inhibits activity of p-eIF2α, and GSK2606414, which inhibits PERK-dependent phosphorylation of eIF2α, both prevent SG formation in the context of sodium arsenite and DTT (Figure 2A). As above, if osmotic stress induces SG formation via the ISR, one expects that both of these compounds would prevent SGs from forming during osmotic stress. The fact that they do not further supports that osmotic stress leads to granule formation via a non-ISR pathway.

While validating that GSK2606414 indeed prevents phosphorylation of eIF2α in our cell culture model, we noticed that all three stressors lead to increased p-eIF2α abundance (Figure 2C; Supplementary Figure S3). GSK2606414 reduces p-eIF2α abundance during sodium arsenite and DTT treatment, but not sorbitol treatment (Figure 2C; Supplementary Figure S3). This supports that sorbitol is leading to phosphorylation of eIF2α via some PERK-independent mechanism. However, combined with the fact that treatment with ISRIB, which prevents translational inhibition upon eIF2α phosphorylation (Sidrauski et al., 2013), does not prevent formation of stress granules, we are hesitant to conclude that this phosphorylation event is entirely responsible for downstream SG formation following osmotic stress.

While investigating the effect of cellular stress on the compartmentalization of polyA RNA, we observed that sorbitol, but neither sodium arsenite nor DTT, leads to a significant reduction in the NC ratio of polyA RNA (Figures 3A,C). Perhaps unsurprisingly, this occurs in both WT and G3BP1/2 KO U2OS cells, as with our other observations. We were unable to determine whether the formation of RNA granules is required for this shift in RNA, as the two stress granule inhibitors we used had little to no effect on SG formation following sorbitol treatment. A future investigation may yield an effective way to prevent SG formation in the context of hyperosmotic stress, which would be useful to establish a causative relationship between sorbitol-dependent RNA egress and granule formation.

Importantly, the possibility of a sorbitol-specific off-target effect is excluded by our observation that other polyols (D-Mannitol and Xylitol) lead to the same shift in polyA NC ratio (Figures 3B,D). We determined that this shift occurs via active mRNA transport, as no difference in polyA NC ratio is observed between untreated and sorbitol-treated cells following NXF1 knockdown (Figure 4). The precise mechanism underlying this shift remains enigmatic, but future investigations will be required to understand whether this shift confers some benefit to cells during osmotic stress, or whether it is merely an indirect consequence of altered intracellular or intranuclear osmolarity. Interestingly, cytoplasmic RNA granules still form following NXF1 knockdown (Figure 4C), suggesting that it is not only newly exported mRNA that gets incorporated into SGs.

Such a striking change in the typical distribution of RNA led us to question whether nuclear RBPs are similarly shunted into the cytoplasm or move within the nucleus following hyperosmotic stress. Since standard immunofluorescence would fail to provide insight into subnuclear movement of proteins (i.e., any proteins that shift between the chromatin-bound or nucleoplasmic pools), we employed a modified fractionation protocol (Conrad et al., 2017) to separate the cytoplasm, nucleoplasm, and chromatin from HeLa cells under various conditions. We observed via Western blot that numerous nuclear RBPs demonstrate a significant change in subcellular localization following hyperosmotic stress, but not oxidative or ER stress (Figures 5A,C). Of the RBPs tested, those that demonstrated a significant response to sorbitol tended to shift to a more cytoplasmic pool, similar to what we observed with polyA RNA (Figure 3). It is possible that the shifting of the RNA and the RBPs is linked, with one “piggybacking” into the cytoplasm with the other. This has been thoroughly demonstrated in the case of TDP-43, whose localization is tightly linked with its mRNA targets (Duan et al., 2021). Whether or not the RBPs we present here are shifting under a similar mechanism is unknown.

Although we observe numerous RBPs deviating from their normal compartmentalization, this does not necessarily mean that the proportion of each RBP that remains in its expected location is unable to perform at full capacity. Protein abundance may be flexible enough to allow for full function even if, for example, 20% of a specific protein is located away from its normal functional locus. Thus, we perform a functional assessment for a subset of the shifted proteins by quantifying changes in their mRNA targets. TDP-43, whose identified targets are numerous, demonstrates no clear loss of function following treatment with any of the stressors (Figure 6A). This is unsurprising, at sorbitol leads to even stronger TDP-43 association with the chromatin than in untreated cells (Figures 5A,C), so one would not expect it to be any less functional than normal. hnRNP I, which is significantly more present in the cytoplasm following osmotic stress, similarly shows no alteration in function. hnRNP U, being the most significantly shifted away from the chromatin fraction, does appear to have reduced function during osmotic stress, as demonstrated by a decrease in *IER3* mRNA abundance (Figure 6B). This provides evidence that osmotic stress is unique in its ability to re-localize RBPs and alter or reduce their normal function, at least within the context of these experiments.

Beyond the fact that hnRNP U appears *more* shifted than hnRNP I, we are surprised that hnRNP I function is not altered under the same conditions. Using 5-bromo-uridine to visualize the amount of nascent RNA produced by cells during stress, we observe that all three stressors lead to significant or trending loss of transcriptional activity (Figures 6C,D). This has recently been observed in the context of KCl-induced hyperosmotic stress (Rosa-Mercado et al., 2021). Although the specific mechanism underlying transcriptional repression in our experiments remains unknown, it is possible that, in the context of sorbitol treatment, the observed cytoplasmic shift of hnRNP U may be responsible. hnRNP U, and its association with actin, is functionally linked to productive transcription by RNA polymerase II, with reduced hnRNP U abundance leading to less transcription (Kukalev et al., 2005). Although the total abundance of hnRNP U is unchanged by sorbitol treatment, this re-localization may lead to the same functional consequence as a true knockdown experiment. Nevertheless, the observed reduction in transcription may “hide” any reduced function of those RBPs that are less shifted from the nucleus than hnRNP U. Since our qRT-PCR assays are dependent on active transcription to pick up differences in the abundance of RBP transcriptional targets, it is now *un*surprising that hnRNP I, which appears to be strongly shifted from the nucleus, may not demonstrate reduced function in the context of osmotic stress.

Given the central role that RBPs play in the pathogenesis of numerous diseases, it has become essential that we understand their normal regulation *and* how they are perturbed in a disease state. Here, we provide insight into the effect of commonly used cellular stressors on the localization and function of several well-known RBPs. Additional proteome- and transcriptome-wide investigations will provide a powerful and unbiased view of the RBP “forest,” while targeted, mechanistic studies such as ours will help us distinguish the RBP “trees.” Together, these sets of investigations will strengthen our understanding of RBP regulation, and aid in the identification of effective targets for the treatment of disease.

## Data Availability

The raw data supporting the conclusion of this article will be made available by the authors, without undue reservation.
