# Supplementary material for: Polyadenylated RNA and RNA-Binding Proteins Exhibit Unique Response to Hyperosmotic Stress
Source: Front Cell Dev Biol. 2021 Dec 14;9:809859. doi: 10.3389/fcell.2021.809859 (PMC8712688; doi:10.3389/fcell.2021.809859)
Supplement: Supplementary file 1 [file DataSheet1.PDF]

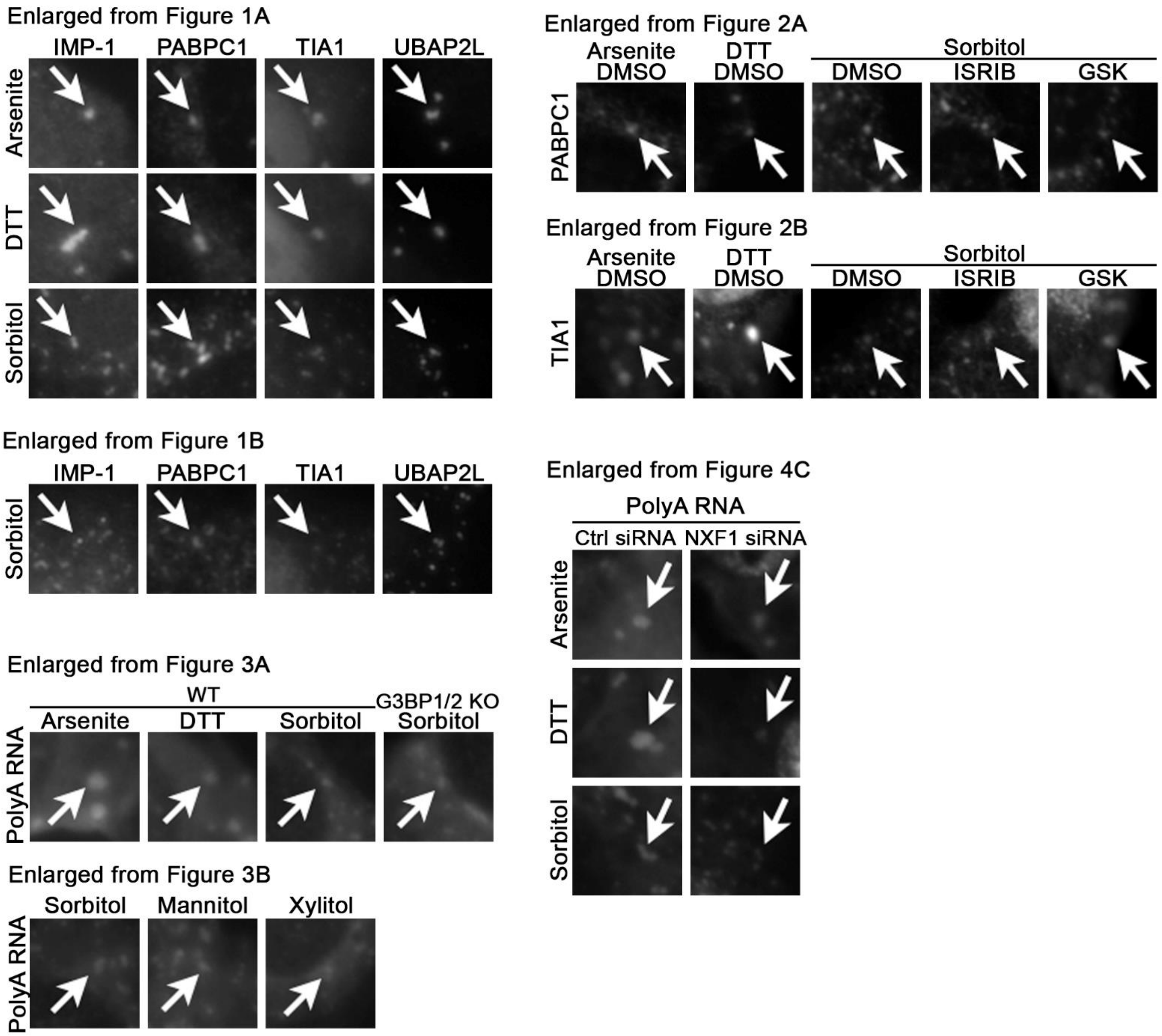

Supplemental Figure 1 (related to Figures 1-4): Enlarged images depicting stress granules
Images from Figures 1-4 that depicted presence of stress granules were enlarged 2.7-fold in each direction to improve the visibility of regions of interest around the arrows in the original images.

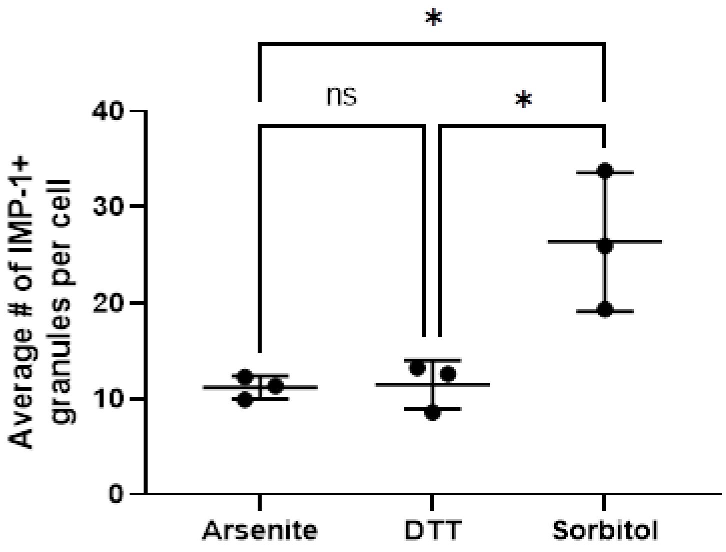

Supplemental Figure 2 (Related to Figure 1): Sorbitol induces formation of more stress granules than sodium arsenite and DTT

The average number of IMP-1-positive granules per cell was calculated using a granule counting module from MetaXPress. N = 3 biological replicates. Ordinary one-way ANOVA with Tukey’s multiple comparisons test was used to calculate statistical significance. *$p<0.05$.

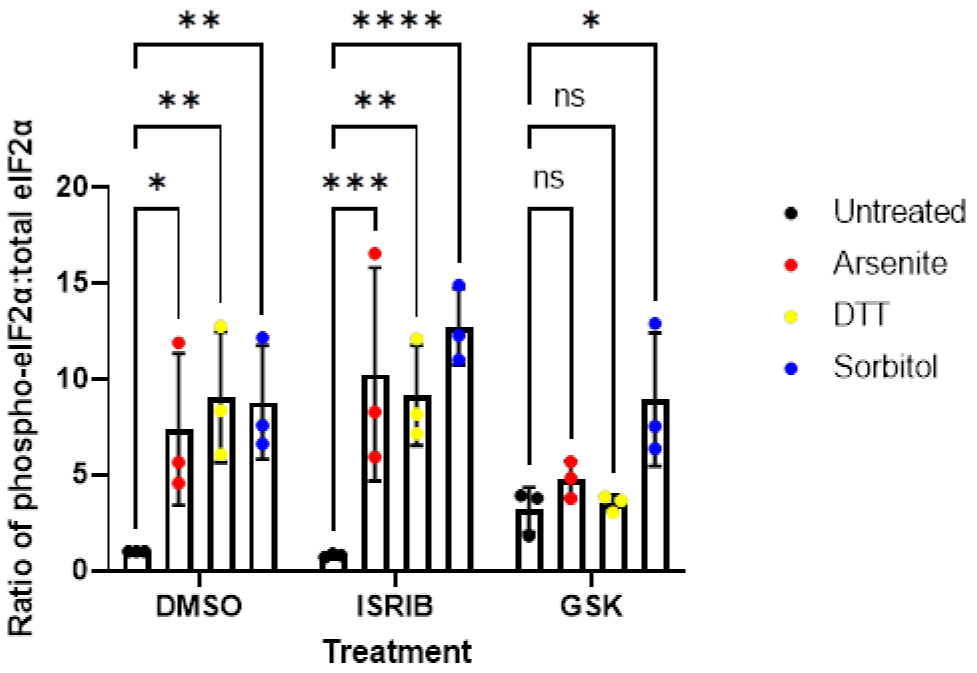

Supplemental Figure 3 (related to Figure 2): Quantification of p-eIF2α:total eIF2α ratio
Quantification of the relative intensity of the phospho-eIF2α and total eIF2α bands represented in Figure 2C. Values presented are the ratio of the raw intensity of the phospho-eIF2α and total eIF2α bands from each corresponding blot. N = 3 biological replicates. Two-way ANOVA with Dunnett's multiple comparisons test was used to calculate statistical significance. *$p < 0.05$, **$p< 0.01$, ***$p< 0.001$, ****$p< 0.0001$.

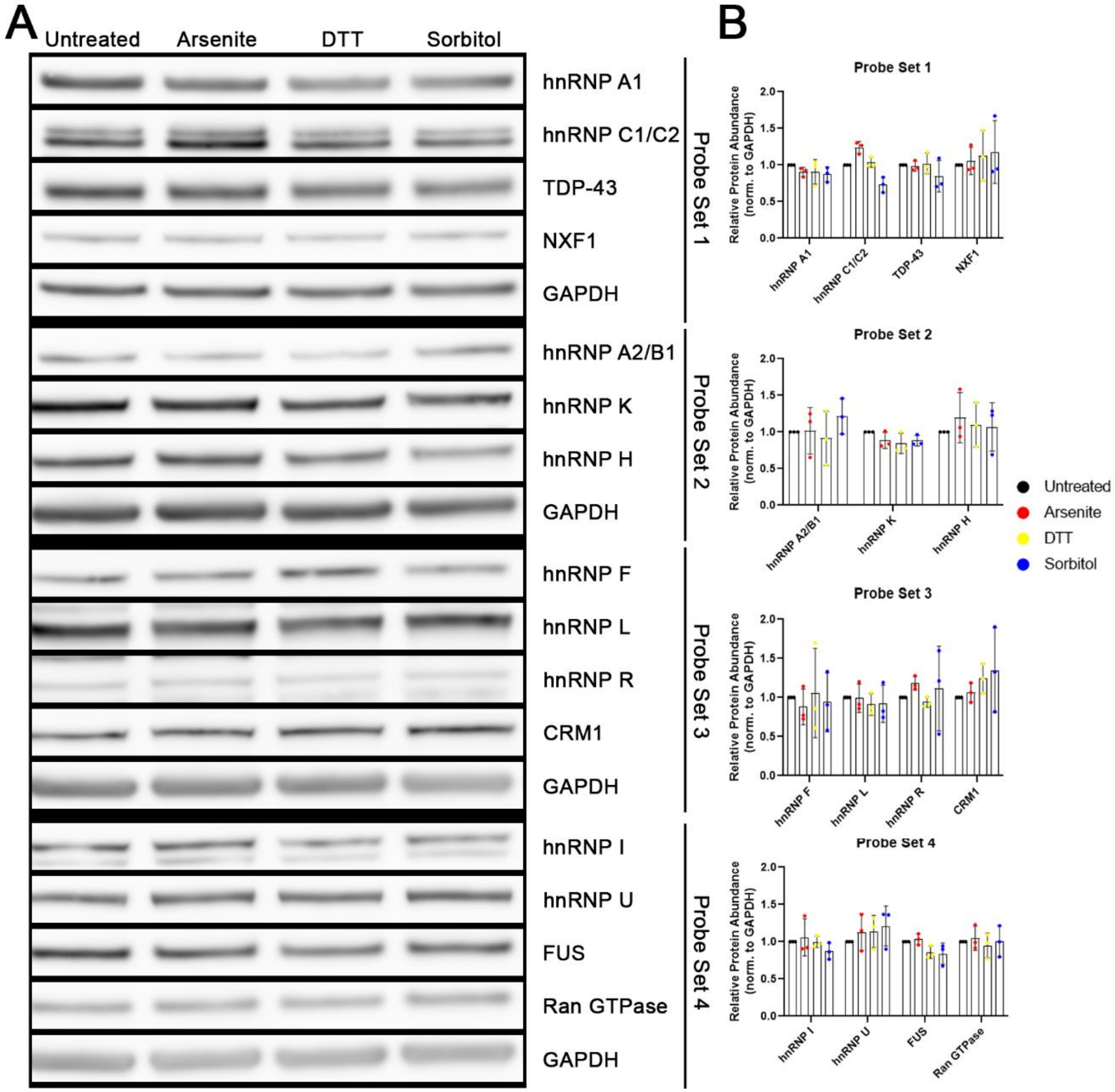

Supplemental Figure 4 (related to Figure 5): Total RBP abundance is not significantly altered by cellular stress

A) Western blots of whole cell lysates from HeLa cells treated with the indicated stressors for 1 hour. Images are all presented from the same biological replicate, grouped based on the probes for each distinct membrane (4 gels/membranes were need for each replicate). Samples from 3 biological replicates were probed for each of the indicated proteins. B) Quantification of the intensity of each band represented in (A), relative to the corresponding GAPDH band from each blot, and normalized to the values from the untreated samples. Two-way ANOVA was used to calculate statistical significance.
